# Influence of Lifestyle and Dietary Habits on the Prevalence of Food Allergies: A Scoping Review

**DOI:** 10.3390/foods12173290

**Published:** 2023-09-01

**Authors:** Gardiner Henric Rennie, Jinlong Zhao, Mukeshimana Camus-Ela, Jialu Shi, Lan Jiang, Lili Zhang, Jin Wang, Vijaya Raghavan

**Affiliations:** 1 Key Laboratory of Environmental Medicine and Engineering, Ministry of Education, and Department of Nutrition and Food Hygiene, School of Public Health, Southeast University, Nanjing 210009, China; henricgardiner@gmail.com (G.H.R.); kinlongzhao@163.com (J.Z.); jiangl59@163.com (L.J.);; 2 Department of Bioresource Engineering, Faculty of Agricultural and Environmental Sciences, McGill University, 21111 Lakeshore Rd, Sainte-Anne-de-Bellevue, QC H9X 3V9, Canada

**Keywords:** food allergy, lifestyle, dietary habits, scoping review

## Abstract

Changes in behavior, lifestyle, and nutritional patterns have influenced many potential risk variables globally. In recent decades, food allergies (FAs) have been elevated to a severe public health issue both in developed countries and developing countries (third-world countries). This study aims to evaluate the effects caused by certain factors such as lifestyle and dietary habits on food allergies, review the association of lifestyle and dietary habit status with FAs, and outline why more people are allergic to food sources as a result of lifestyle changes and dietary habits. We searched electronic international databases including Scopus, PubMed, Google Scholar, and Web of Science using combinations of keywords. Utilizing Excel, the relevant studies were included and the irrelevant studies were excluded, and Mendeley was used for referencing and also to remove duplicates. The framework proposed by Arksey and O’Malley was used for this scoping review. The papers published in the databases from 2016 to 2020 were extracted. A total of eight studies were extracted, and this scoping review was carried out according to the risk factors. In our review, we found that some lifestyle choices (Caesarean section and antibiotics) and dietary habits (n-3 PUFA, fast food, duration of dietary intervention, and vitamin D), were important contributing factors for FA.

## 1. Introduction

In the past decade, food allergies (FAs) have emerged as an important public health concern both in developed (industrialized) and developing countries [1]. This issue has raised concerns among various stakeholders, including patients and their families, healthcare providers, schools, food producers and merchants, and government agencies [2]. FA is an early manifestation of allergic disease that occurs due to abnormal immune responses to antigens, leading to sensitization. Sensitization happens when the immune system produces antigen-specific IgE (sIgE) in response to allergenic food proteins. Upon re-exposure, the allergenic food binds to specific antibodies, triggering the release of mediators like histamine, resulting in symptomatic FA [1]. Furthermore, sensitization can occur on other surfaces, which include the skin, with allergen exposure in the microbiota (gut) leading to allergic reactions [3]. Allergy symptoms usually appear a few minutes after eating, but can sometimes take up to hours to appear. The symptoms of food allergy in various parts of the human body can range from mild to severe (anaphylaxis) [4]. There is still no cure for some food allergies; thus, allergic patients are advised to eliminate allergens from their diets [5]. However, the only life-saving treatment for food-induced anaphylaxis is epinephrine [6].

**Figure 1:**
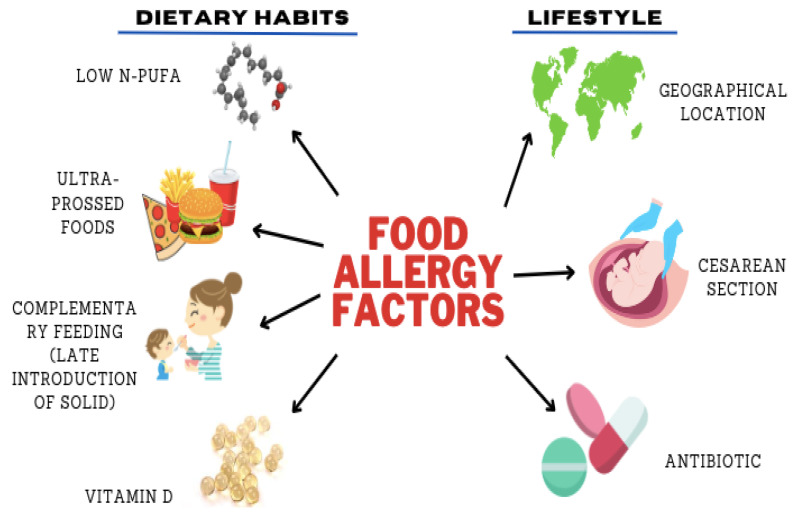
The factors responsible for food allergies.

Several studies have indicated a global increase in the prevalence of food allergies (Figure 1), not only in Western nations but also in regions influenced by a Western lifestyle [7]. Despite advances in our understanding of the disease, many fundamental questions about food allergies remain unanswered. Specifically, the reasons for the increasing prevalence, the demographics most susceptible to developing food allergies, and the explanation for the heightened vulnerability of young children to this condition are still unknown. Determining the factors responsible for food allergies presents several challenges. Publications on the prevalence of food allergies exist, but it is uncertain whether these studies provide accurate estimations or precise information. As a result of the limited quantity of studies, our knowledge about the factors driving food allergies in older adults is limited. However, food allergies are widely observed in young children in industrialized countries, and recent data suggest increasing occurrence among teenagers and young adults, even in underdeveloped nations [7,8]. Yet, the lack of a standardized definition makes it difficult to quantify the true global burden of food allergies among children and adults. Recent data reveal that approximately 8% of children are affected by food allergies, with around 2.4% experiencing multiple sensitivities and up to 3% encountering anaphylactic reactions [9].

The gold standards of diagnosis, such as placebo-controlled food challenge (DBPCFC) and double-blinding, have only occasionally been utilized in a few studies to obtain the prevalence of FA. In contrast, much research has solely relied on participants’ subjective perceptions of food reactions, commonly referred to as self-reported food allergies (SRFAs). When compared to research that employed objective diagnostic techniques, the majority of these studies seem to have exaggerated the prevalence of FAs [2]. Despite food allergies being recognized for over a decade, there remains a lack of comprehensive analysis regarding the risk factors. However, the rapid increase in the occurrence of this issue suggests that lifestyle modifications, rather than genetic predisposition, may contribute to the rising burden of allergic diseases. Ribeiro et al. [10] further affirms the significance of diet in human health, and recent shifts towards unhealthy dietary consumption have already been correlated with the rise of numerous non-communicable diseases.

The interconnections between modifiable and non-modifiable risk variables are influenced by changes in behavior, lifestyle, and eating habits [11]. Figure 1 illustrates several factors, including fatty acids, fast food consumption, Caesarean section, antibiotic exposure, timing and route of exposure, geographical location, and vitamin D exposure, that have been proposed as contributing to the concerning increase in FA occurrence [12,13]. However, this topic remains contentious. Understanding the natural course of food allergies is crucial as it aids in diagnosing and determining when FA therapy should be considered or administered [14,15]. This scoping review seeks to investigate the relationship between the up-to-date literature on lifestyle and dietary habits and their association with FA in adults and children/infants. Our focus is particularly on the factors contributing to the rise in FA prevalence in both developing and developed nations worldwide. To our knowledge, this is the first scoping review and meta-analysis specifically addressing the impact of lifestyle and dietary habits on FA in young adults, infants/toddlers, and older individuals.

### 1.1. Clinical Manifestation/Symptoms of Food Allergy

**Figure 2:**
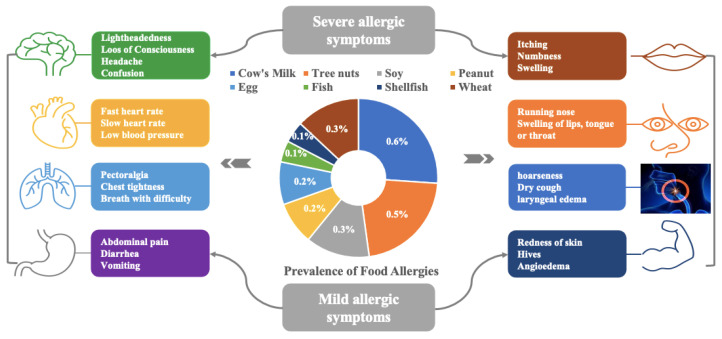
Prevalence of food allergies for common food allergens and the mild and severe reactions to food allergies.

The majority of the time, allergy symptoms emerge shortly after eating, but they can occasionally appear up to 0–2 h after eating for IgE-mediated allergies and 4–72 h after eating for non-IgE-mediated allergies, Figure 2.

Certain organs, including the skin (local or systemic urticaria, atopic eczema), gastrointestinal tract (abdominal cramps, diarrhea, vomiting), and respiratory tract (including the nose and lungs), can exhibit typical signs of systemic responses. Anaphylactic shock affects the cardiovascular system, in addition to allergies and asthma [16,17]. Immediate food sensitivity can affect several organ systems, cause life-threatening hypovolemic shock, and harm to the respiratory system in its most severe form (known as anaphylaxis) [4]. Contact with problematic foods may result in adverse reactions such as sneezing, nasal congestion, coughing, shortness of breath, and wheezing from respiratory illnesses; itching and angioedema from oral allergy syndrome; nausea, vomiting, and diarrhea from gastrointestinal diseases; and urticaria and angioedema from skin diseases. Additionally, food-related anaphylaxis and fatalities have been reported [18]. Food allergy symptoms can manifest in many areas of the body. Based on the distinction between IgE-mediated and non-IgE-mediated allergic reactions, symptoms are categorized.

### 1.2. Pathophysiology/Mechanism of Food Allergy(ies)

The reaction to ingested antigens can be triggered by a variety of immunological processes associated with food allergies. Understanding how food allergens trigger allergic immune responses is necessary before we can comprehend the mechanisms of food allergy. The common natural dietary proteins of both plant and animal origin are the source of food allergies [19].

### 1.3. Exposure Route

Contrary to the path that results in allergy sensitization, tolerance to possible allergens is a possibility. Tolerance can be achieved by inhibiting several regulatory cell populations, the most prevalent of which are the following regulatory T cell subsets (Treg cells), or by lymphocyte anergy. Forkhead box protein P3 (FOXP3)-expressing CD4+ T cells and IL-10-producing CD4+ T cells, also known as FOXP3-CD4+ 1 type regulatory T cells (TR1), are both types of CD4+ T cells [20]. Age, mucosal barrier state, genetics, the physical characteristics of the antigen, dose, gut flora, and frequency of exposure all play a role in oral tolerance improvement [21]. Rather than causing oral tolerance in susceptible people, exposure to dietary protein causes allergy sensitization [18]. Proteins from food are taken up by dendritic cells after they penetrate the mucosal barrier and are sent to the neighborhood nymph ganglia. Through class II molecules of the major histocompatibility complex on the surface of dendritic cells, these ingested proteins are degraded into peptides and given to naive CD4+ T cells. The cytokines [i.e., interleukins (IL) IL-4, IL-5, IL-9, and IL-13] are produced by the native T cells in TH2 cells as a result of this process. In turn, the TH2 cells employ IL4 and IL13 to induce the IgE class transition and activate naive B cells. Activated B cell clones with a strong affinity for antigens can grow even more when exposed to TH2 cells. To further stimulate the activation of TH2 cells, activated B cells can serve as antigen-presenting cells. High-affinity IgE receptors (FcRI) on mast cells and basophils bind to IgE molecules generated by activated B cells. Re-exposure to the allergen causes the IgE molecules on the granulocytes to crosslink, which results in degranulation. Granulocyte degranulation causes vascular permeability to rise, the release of inflammatory mediators, constriction of the airways, and the recruitment of more inflammatory cells [20]. An acute phase reaction may result from the subsequent release of mediators (histamine, cytokines, inflammatory lipids, and chemokines) [21]. Following the acute phase response, eosinophils and TH2 cells are attracted to the reaction site by chemokines produced by mast cells. Mast cells and eosinophils are triggered by the IL-5 and IL-9 generated by TH2 cells, and they release extra mediators and have delayed reactions [18].

### 1.4. Diagnosis of Food Allergies

Clinical history is possibly the most crucial “test” for determining a food allergy. This history must be evaluated in light of the knowledge of the clinical manifestations and epidemiology of food allergies as well as with an understanding of illnesses with comparable clinical presentations that can be mistaken for food allergies to fine-tune a diagnosis. See Table 1 below [6,22]. Once the suspected food allergen has been established, first-line diagnostic testing involves either measuring the serum food-specific IgE levels, doing a skin test, or both. IgE antibodies to food (also known as sensitization) are found through the measurement of serum food-specific IgE. Using extracts and a tool to itch or puncture the skin, an allergen is injected into the epidermis during a skin test. Sensitization is indicated by the appearance of a wheal and flare. In the diagnosis of IgE-mediated food allergies, both testing methods are quite sensitive. For skin tests, the sensitivity is greater than 90%, and for serum food-specific IgE measurement, it ranges from 70% to 90% [6]. The only reliable test for determining if a person has a food allergy is the OFC, which involves consuming progressively higher doses of a putative allergen over a set period until either an allergic reaction is noticed or a maximum dose is reached. This diagnostic technique, however, is rarely performed outside of specialist institutes that are adequately staffed and equipped because it is resource-intensive and runs the risk of causing a severe allergic reaction. A referral mechanism should ideally be in place in a clinical context where food allergy SPT and sIgE tests are performed to establish a definitive diagnosis utilizing OFC [23].

### 1.5. Management and Prevention of Food Allergies

In the absence of treatment, the successful management of food allergies necessitates complete abstinence from the allergen-causing food. Avoiding allergens is the most secure method of managing the FA [24]. As a first-line treatment for food-induced anaphylaxis, injectable epinephrine should be given to all patients with a food allergy due to the inability to accurately and consistently forecast the severity of future allergic reactions [25]. The International Life Sciences Institute (ILSI) advises that to provide accurate information about dietary restrictions and a comprehensive list of allergens known to be linked to cross-reactivity, dietitian advice should be sought [26]. Contrarily, regulatory bodies have decided to concentrate allergy labeling rules on a small number of priority allergens, even though more than 200 food items are allergenic. The Food Allergen Labeling and Consumer Protection Act (FALCPA), approved by the US Congress in 2004, mandates that any item containing a component that is or is a derived protein of a “major food allergen” mention the allergen’s presence on the label in the way required by law [27]. Education about illnesses, food allergies’ natural history, available treatments, how to read labels, and how to prevent unintentional exposure or intake should be given to parents or patients [28]. Although the Australian Society of Immunology and Clinical Allergy, the European Society of Pediatric Gastroenterology, Hepatology, and Nutrition, and the American Academy of Pediatrics have all recommended avoiding food allergens during pregnancy and lactation, some studies have now found that early exposure to potential food allergens can be beneficial [4]. Consuming common food allergens including peanuts, tree nuts, milk, and wheat while pregnant may reduce the likelihood that infants will develop food allergies. In a similar vein, giving babies a wider variety of foods throughout infancy may be associated with a decreased risk of allergic sickness [23].

## 2. Materials and Methods

### 2.1. Study Design

**Figure 3:**
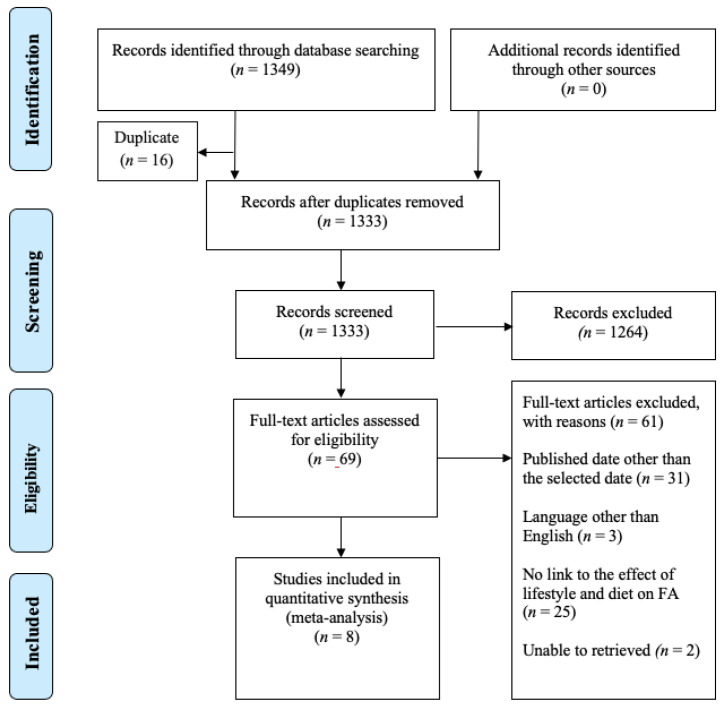
Flowchart diagram: the process of identification and selection of studies.

Our report outlines the methodological approach used in guiding a scoping review. “A scoping review, also known as a scoping study, is a type of statistical synthesis that carefully examines, selects, and synthesizes existing knowledge to address a research question that aims to map the main concepts, types of evidence, and research gaps pertinent to a certain subject or field” [29]. In summary, we established a pre-defined framework for the review process [30] that included five steps: identifying the research question; identifying relevant studies; study selection; charting the data; and collating, summarizing, and reporting the results as illustrated in Table 2 below. To guide our research, we utilized an outline proposed by Arksey and O’Malley [31] that was further advanced by Levac et al. [32]. Additionally, we employed the Joanna Briggs Institute (JBI) methodology [29] and followed the Preferred Reporting Items for Systematic Reviews and Meta-Analyses (PRISMA) guidelines [29,33]. By utilizing Microsoft Excel, studies relevant to our research were included, and those that show no correlation were excluded. Mendeley was used for referencing as well as to remove duplicates. The excluded raw data file is present as the supplementary file. Figure 3 presents a flowchart depicting the study selection technique, along with an evaluation of the methodological quality of the primary research.

### 2.2. Research Questions

The following research questions were proposed:

What insight does the literature offer about the correlation between lifestyles, dietary habits, and the prevalence of FA?

What are lifestyle and dietary habit factors that might lead to FA?

### 2.3. Source of Data/Studies Identification

A multidisciplinary team with experience in the field executed a thorough and highly sensitive search strategy in prominent databases with no geographical limits.

We conducted an electronic search utilizing the terms “prevalence”, “FA”, “lifestyle”, and “dietary habits” in the listed electronic international databases, Google Scholar, Web of Science, Scopus, and PubMed, with only one language constraint (English language). As seen in the example, the keywords were utilized separately and in combination as a syntax employing boolean operators such as “AND”, “OR”, and the “*” symbol (Table 2). The search approach was created for Medline and then expanded to include the other databases. The search was restricted to recent studies, defined as those published within the last five years (between 2016 and 2021). Other relevant materials were discovered by looking through the references of the selected articles.

### 2.4. Studies Selection

Table 3 presents the key criteria used for including and excluding articles in our study. Initially, we employed the search approach described earlier to identify all potential research articles. Two authors conducted a subsequent evaluation of the titles and abstracts of the identified publications. Any publications that were identified as potentially relevant underwent a comprehensive analysis of their complete texts to determine their eligibility. In cases where there were disagreements between the two authors regarding the assessment of a study, a third author, who possesses significant experience in review research, was consulted to resolve the matter.

### 2.5. Data Charting

The selection of the eligible literature took into account information such as author(s), year of publication, nation (country), study sample size, design of the publication, study (research) limitation(s), and the outcome significant to the study questions mentioned.

### 2.6. Results Collation, Summary, and Report

A comprehensive study was conducted to address the two research questions that were formulated, ensuring the inclusion of their essential components. The quality of the included studies was assessed using the Joanna Briggs Instrument Critical Appraisal Checklist [30]. The outcomes of all of these studies were analyzed and contrasted until the primary themes emerged. Utilizing a tailored data extraction form in Microsoft Excel, we marked all the frequency estimates of food allergy occurrence to assess the total prevalence of lifestyle and dietary habits. The findings of the study are presented in a descriptive format. Our main factor of interest was specifically included in the calculations for research providing more than one risk factor. Table 4 provides important details along with a basic numerical analysis that highlights the characteristics and distribution of the research (studies) considered in the scoping review. It explicitly presents information about the groups studied, the methodologies employed, and the main conclusions drawn from the works.

## 3. Results

### 3.1. Study Characteristics

The searches identified a total of 1349 records; all citations were organized in a Mendeley reference/desktop manager. After eliminating duplicate records, the search of electronic databases and review article references yielded a total of 1333 citations. The findings are displayed in a PRIS-MA-ScR flow diagram. Based on the evaluation of titles and abstracts, we excluded 1264 records that did not meet the inclusion criteria. Subsequently, 69 full-text articles were obtained for further assessment of their eligibility. Of these, 60 were excluded for the following reasons: 31 of the 60 papers were published out of the time limit selected for this scoping review, which is 2016 to 2020; three were in language order than English (other languages such as Spanish, Portuguese, Chinese, and Russian); and 25 did not directly quantify the effects of lifestyle and dietary habits on the prevalence of FA. Two studies were excluded because we were unable to retrieve them. The remaining eight studies were considered eligible for this review, as shown in Table 4.

The majority of the studies (*n* = 8) were undertaken among children with one in the adult population, usually those 34 weeks to 27.9 years of age. Of the few studies undertaken in this scoping review, there was very little research conducted in North America, Southern Africa, Southeast Asia, and Southern Oceania, compared with research from Southern, Northern, and Western Europe. Table 5 illustrates the odds ratio in each of the selected studies.

Every single evaluation method (SPT sensitization, specific IgE sensitization) and self-reporting was utilized to quantify FA for each FA; however, self-reporting was the most usually used method. To quantify FA, a few studies paired symptoms with either SPT or IgE sensitization. Excluding the initial sample size of the research, participation ranged from 102 to 1,001,294. For each of the factors studied, there were also more reports based solely on self-reported FHS than those that included an objective test to assess FA. The features of the studies considered in the review are listed in Table 4.

### 3.2. Effect of Dietary Habits on FA

#### 3.2.1. Fatty Acid/Polyunsaturated Fatty Acid (PUFA)

One of nine studies involving 250 pregnant women aged 19 years and above from the City of Johannesburg in Gauteng, as well as those attending ANC at the Rahima Moosa Mother and Child Hospital (RMMCH) in South Africa, looked at the links between maternal dietary intake and nutritional status markers of neonatal exposure and the chance of developing FA and allergic diseases in a cross-sectional manner at 18 weeks of pregnancy. The reduced consumption of n-3 polyunsaturated fatty acid (PUFA) and increased consumption of n-6 PUFA are dietary-associated risk factors for allergic illness, according to the study, with an odds ratio (OR) of 1.21 and a 95% CI of 1.10, 1.33. According to the ISAAC survey, 19.6% of the women reported having an allergy [36].

#### 3.2.2. Ultra-Processed Foods (UPFs)

In one of the included studies, the relationship between dietary patterns and food allergies (FAs) was assessed using the International Study of Asthma and Allergies in Childhood (ISAAC) questionnaire. This study involved a population-based sample of 3744 Australian children who participated in the cross-sectional Mothers and their Children’s Health (MatCH) project. The findings revealed that children following a “Sweet and savory snacks” pattern had a slightly higher likelihood of developing food allergies compared to those with a lower intake of snacks. The authors concluded that allergic diseases are prevalent among Australian children, with 36.9% of the sample experiencing at least one allergy. This increased prevalence of allergies could potentially be associated with a higher frequency of consuming non-core foods, particularly within the realm of food allergies [10].

Similarly, the connection between FA and specific food consumption frequency (fast meals, energy drinks, or convenience food) in adolescents was explored in 53,373 South Korean individuals. However, when examining UPF with a weight of 18.23%, it can be concluded that the study demonstrates an elevated risk for FA. Ultra-processed foods (fast foods), energy drinks, and convenience food consumption were considerably higher among those with recently diagnosed FA. Fast food consumption was considerably higher among patients with newly diagnosed food allergies, OR 1.40 (95% CI 1.15, 1.72) [39].

#### 3.2.3. Late Introduction/Exposure to Solids

One study used a cross-sectional observational study design with a total sample size of 1029 children aged 7 to 8 years old in two geographic locations in Sweden, Mölndal in the southwest and Kiruna in the north, to look into the link between late solid introduction or exposure and the risk of developing food allergies. The cumulative incidence of self-reported FA or intolerance (SRFA) was 19.6%, with Kiruna (28.5%) having a substantially higher cumulative incidence than Mölndal (15.7%), *p*.001. The later introduction (7 months) of solids could be one factor for the greater cumulative incidence of SRFA in Kiruna [38].

#### 3.2.4. Vitamin D Insufficiency

A study conducted in Finland investigated the key factors influencing vitamin D status in Finnish school-aged children, with a focus on the history of FA diseases. This study included 171 ten-year-old participants predominantly of Caucasian heritage. The ISAAC questionnaire was utilized to assess parent-reported symptoms or physician diagnosis, following the Helsinki and Uusimaa Hospital District Ethics Committees’ criteria. The findings revealed that 16% of those who took vitamin D supplements 2–7 times per week had a vitamin D deficiency (defined as <50 nmol/L) compared to those who did not take any or only once a week (OR 0.14, 95% CI 0.04, 0.24). Moreover, children with a history of cow’s milk allergy exhibited lower 25(OH)D levels than those without an allergy (60.5 ± 12.6 nmol/L vs. 75.5 ± 22.3 nmol/L, *p* = 0.004). The study also indicated a link between lower vitamin D levels and infrequent intake (once a week or less, or never) of vitamin D supplements, as well as a diagnosis of cow’s milk allergy [35].

### 3.3. Effect of Lifestyle on FA

#### 3.3.1. Geographical Locations/Racial Disparities

A UK cross-sectional study with a sample size of 3300 children aged 3 to 16 looked at the relationship between cultural differences in nut consumption during pregnancy, breastfeeding, and early childhood exposure to nuts and the reported prevalence of nut allergy in three populations: Libyans, UK Libyans, and the general UK population. The study sample had a total prevalence of 3.1 percent for peanut allergy (35 per 1123 cases). The odd ratio, OR, was 1.07 (95% Cl 0.11, 10.35) for the Libyan and UK Libyan populations, making them nearly equivalent. Through tolerance induction, continuous and early moderate nut intake throughout childhood as well as maternal ingestion during pregnancy or lactation may help in avoiding the development of peanut and almond allergy [40].

#### 3.3.2. Caesarean Section

One of the nine studies with a total of 459 children born at ≥34 weeks of pregnancy and cared for in a tertiary maternity division were scrutinized at birth and followed up with at 1, 6, 12, 18, 24, 30, and 36 months of age in Greece in a prospective birth cohort study that studied the relationship between C-section and physician-diagnosed FA, evaluated via skin prick tests (SPTs) and/or blood sampling for serum-specific serum immunoglobulin IgE (sIgE) detection. Children born through CS to at least one allergic parent had a greater risk of developing food allergies than children born vaginally to non-allergic parents [34].

#### 3.3.3. Antibiotic

Antibiotic nonusers were matched 1:1 to antibiotic users on date of birth, sex, race, and state in one of the studies included. Antibiotic exposure is related to a somewhat increased risk of FA development, with a weight of 25.26% OR 1.40 (95% CI, 1.34, 1.45). There was a substantial link between antibiotic exposure and the onset of food allergies [37].

### 3.4. Principal Findings

Identifying the breadth of available evidence to better understand how changing environmental factors affect diet and lifestyle during adolescence and early adulthood is a critical first step in informing further evidence synthesis and epidemiological analysis, as well as providing evidence to support the appropriate targeting of lifestyle and dietary public health interventions. The goal of this scoping review study is to look into the link between lifestyle factors and dietary habits and FA in adults and children/infants. To our knowledge, this is the first overview of a scoping review on the coverage of the effect of lifestyle and dietary habits as it relates to FA in adults and children/infants. The research (*n* = 8) involved children, with one adult study involving persons aged 34 weeks to 27.9 years. Only a few studies in North America, Southern Africa, Southeast Asia, and Southern Oceania were included in this scoping review, compared to studies from Northern Europe, Southern Europe, and Western Europe.

#### 3.4.1. n-3 PUFA (Polyunsaturated Fatty Acid)

Environmental exposures in childhood are powerful predictors of health and disease later in life, according to epidemiological studies. One major exposure that affects early development and later consequences has been identified as nutrition. The human immune system develops significantly throughout pregnancy and in the weeks and months following delivery, and there is evidence that nutritional variables might influence early immunological development. The reduced consumption of n-3 PUFA is a dietary-associated risk factor for allergic illness. The consumption of n-6 PUFA-rich meals has increased in recent decades, while consumption of n-3 PUFA-rich foods has dropped. Changes in fatty acid consumption may cause the T-helper balance to change from type 1 to type 2, increasing the risk of IgE-mediated allergy disorders [34]. Our findings, however, are in line with those of other studies. According to a separate study, the Western diet, which is defined by a high intake of processed foods high in fats and carbohydrates, is linked to a disruption of the gut microbiota’s homeostatic equilibrium. These characteristics have a temporary impact on the newborn gut microbiota and thus immune system maturation and allergy development during the prenatal and postnatal periods [41]. A meta-analysis discovered a protective correlation between increased prenatal n-3 LC-PUFA or fish intake and the incidence of allergy illness symptoms in children. The prevalence of “atopic eczema,” “any positive SPT [skin-prick test]”, “sensitization to egg”, and “sensitization to any food” was significantly lower in the first year of life, according to the pooled data (RRs (95% CIs) of 0.53 (0.35, 0.81), 0.68 (0.52–0.89), 0.55 (0.39–0.76), 0.55 (0.39–0.76), and 0.59 (0.46, 0.76. The results of this meta-analysis are suggestive of the advantages of increasing n-3 LC-PUFAs in the maternal diet in terms of pediatric allergy illness outcomes [42]. Another meta-analysis revealed that taking supplements of long-chain n-3 fatty acids while pregnant significantly decreased the probability of the baby developing asthma or wheezing (RR 0.62 [95% confidence interval 0.34–0.91], *p* = 0.005, I2 = 67.4%). No impacts of supplementation on any outcome were seen during nursing in infants [43]. Although the strength of the evidence is weak, taking supplements of long-chain n-3 fatty acids while pregnant may lower the risk of asthma and/or wheezing in the fetus.

#### 3.4.2. Ultra-Processed Foods (UPFs)

Ultra-processed foods, in our opinion, are mass-produced foods that are prepared and served quickly but have poor nutritional value. The consumption of “quick foods” has increased as a result of the acceptance of Westernized lifestyles. Ultra-processed meals are cuisines prepared in restaurants or stores with preheated or precooked components and supplied to consumers in a packaged form for takeaway. They were first popularized in America in the 1950s. UPFs, so-called fast foods, are often heavy in refined carbohydrates, sodium, sugar, cholesterol, and chemicals such as preservatives and colorants, with high saturated fat concentrations [44].

Wang et al. identified substantial links between fast food intake and allergies in a recent systematic review and meta-analysis. The findings were similar across diverse definitions of allergy, as well as severity levels, and revealed a dose–response connection (days per week of consumption). Burger eating was singled out as a specific meal type contributing to the link (rather than other forms of fast food or high-energy drinks) [44].

Furthermore, when ultra-processed meals are consumed regularly, a poor-quality diet develops, resulting in vitamin deficiencies that are likely to contribute to the development and progression of allergies.

Even though UPF has been around for a while, it is less understood how it causes allergies or food sensitivities. However, numerous studies have revealed a correlation between poor diets and a higher risk of developing chronic diseases. Inflammation in the body can be increased by poor-quality diets [45]. When regularly consumed, certain food additives found in ultra-processed meals (such as emulsifiers and artificial sweeteners) can also exacerbate gut inflammation by altering the gut microbiome [46]. Food dyes, particularly cochineal red and tartrazine, as well as the preservatives sodium benzoate and sodium sulfite, may be linked to the production of proinflammatory cytokines, which are crucial for the emergence of allergies, according to some research. Studies on human subjects have also discovered a link between histamine generated by human basophils and food additives like tartrazine and sodium benzoate. One of the molecules that sets off allergy symptoms like wheezing in the chest and shortness of breath is histamine [45]. In response, the body instructs our immune cells, including white blood cells, to combat any invasive infections (such as bacteria or viruses). Theoretically, ultra-processed foods induce more inflammation because the body perceives them as foreign—much like invading microorganisms. As a result, the body launches an inflammatory response known as “fast food fever”. As a result, this causes an increase in inflammation throughout the body [46].

#### 3.4.3. Late Introduction/Exposure to Solid Foods

Food allergies in children and teenagers can have a wide range of outcomes, from minimal sensitivity to the risk of serious complications. Early and late exposure to food allergens in children and teenagers, respectively, are frequently examined as the most significant ways for preventing food allergies [47]. The total pooled lifetime self-reported prevalence for the timing of food when looking at late introduction/exposure of solids was 19.6% in the included paper; this finding is in line with a wide body of experimental data from a variety of research that links late enteral antigen exposure to FA. Delaying the introduction of cow milk products and other food products was linked to a higher risk of eczema and recurrent wheezing in this KOALA cohort birth research from the Southern Netherlands. Postponing the introduction of other food products was even linked to a higher risk of atopic sensitization [48]. Another cohort study from Finland discovered that delayed solid meal introduction was linked to a higher incidence of allergy sensitization to food and inhalant allergens [49]. Early contact with key food allergens between the ages of four and six months is more effective in reducing the formation of food allergies in children and adolescents. According to a meta-analysis, infants who are introduced to eggs earlier (before 6 months of age) have a lower risk of developing an egg allergy than those who are introduced to eggs later (after 6 months of age) (RR = 0.60, 95% CI 0.46–0.79). The subgroup analysis of the six egg studies included in the meta-analysis showed that introducing eggs before the age of six months was associated with a lower risk of developing egg allergy (RR = 0.55, 95% CI 0.40–0.75), and introducing raw eggs and small amounts of eggs (equivalent to weekly protein 0–4 g) were also associated with a lower risk of developing egg allergy (RR = 0.55, 95% CI 0.36–0.85). Additionally, compared to supplementing before 4 months of age, supplementing with eggs within 4–6 months decreased the likelihood of developing an egg allergy (RR = 0.58, 95% CI 0.43–0.78) [50]. Early exposure to food allergies is especially advantageous for children in this regard. When compared to late consumption of solids, it can significantly boost the immune system, reducing the risk of future difficulties.

#### 3.4.4. Vitamin D

Vitamin D is naturally synthesized in the skin, but it can also be obtained through dietary sources. Recent research has focused on exploring the impact of vitamin D on the prevalence and severity of food allergies (FAs). One study conducted in vitro found that the expression of a receptor for advanced glycation end-products (RAGE) was decreased in human umbilical vein endothelial cells when stimulated with calcitriol, a form of vitamin D. This finding suggests a potential inverse relationship between exposure to sunlight, levels of vitamin D, and the occurrence of FA [51]. Collectively, these studies provide strong evidence supporting a connection between the consumption of advanced glycation end-products (AGEs) and the development of allergic disorders, specifically food allergies.

According to recent research, higher vitamin D levels in the blood are inversely related to the likelihood of food allergies. Vitamin D deficiency not only compromises the integrity of the gastrointestinal epithelial barrier and undermines immune cell encounters with food protein antigens, but it may also increase susceptibility to gastrointestinal infection. This ultimately leads to the compromised integrity of the gastrointestinal epithelial barrier and further undermines tolerogenic encounters between immune cells and food protein antigens [52]. The potential link between vitamin D and FA was initially discovered through an indirect observation in a 2007 study. This study revealed a higher number of epinephrine auto-injector prescriptions in northern areas compared to southern areas of the United States. It was hypothesized that this discrepancy in prescription rates could be related to sunlight exposure, a crucial factor in the production of vitamin D. After controlling for various factors, this suggests a possible association between vitamin D and FA [53]. An Australian population-based cohort study with a sample of 5276 one-year-old children underwent skin prick testing for peanut, egg, sesame, cow’s milk, or shrimp allergens to evaluate the effect of vitamin D levels in infantile FA. Infants with vitamin D deficiency were more likely to have several food allergies (≥2) than those with a single FA among those with Australian-born parents [54].

The meta-analysis that included 5105 children conducted subgroup analyses depending on various cutoffs of the 25(OH)D levels (20 versus 30 ng/mL). In the one study that employed 30 ng/mL, it was discovered that children with a 25(OH)D status of <30 ng/mL were more likely to report having a food allergy than children with a status of ≥30 ng/mL (OR 2.04 [95% CI, 1.02–4.04]; *p* = 0.04) [55]. Another meta-analysis looked into the connection between vitamin D deficiency and food allergies. Vitamin D deficiency was found to increase the likelihood of food allergy episodes in children by 68% (adjusted pooled OR: 1.68, 95% CI [1.25–2.27], *p*-value: 0.001). They had a four-times-higher chance of presenting a food allergy episode in their second year of life (adjusted pooled OR: 4.06, 95% CI [1.93–8.56], *p*-value: 0.001) and a 56% higher chance of developing food sensitization (OR: 1.56, 95% CI [1.15–2.11], *p*-value: 0.004). Egg sensitization was nearly four times more likely to occur in Australian children with vitamin D deficiency (adjusted OR: 3.79, 95% CI [1.19–12.08], *p*-value: 0.024). Peanut sensitization was nearly twice as common in children with insufficient vitamin D (OR: 1.96, 95% CI [1.08–3.57], *p*-value: 0.028). Food allergies seem to be more common, especially in the second year of life, when maternal vitamin D levels are lower and infants have insufficient vitamin D levels [56].

#### 3.4.5. Geographic Locations and Racial Disparities

In terms of geographic locations and racial differences, our study discovered that the reported prevalence of peanut allergy in both the study sample and the control group is in line with similar cohort research in Australia, which included 57,000 children and used similar techniques, finding that Asian children born in Australia had a greater frequency of nut allergy than Asian children who had immigrated to Australia, with a prevalence of 3.1% parent-reported nut allergy (2.7% to peanut and 1.7 percent to tree nuts) [57]. A meta-analysis with 3,318,608 participants involved aimed to relate geographic differences with the prevalence of FA. According to the findings, the prevalence of FA was 4.3% overall (95% CI: 0.038–0.047). FAs were more common in America (3.2%, 95% CI: 0.024–0.041), Asia (4.2%, 95% CI: 0.033–0.051), Europe (4.8%, 95% CI: 0.037–0.060), Oceania (7.5%, 95% CI: 0.052–0.102), and Africa (1.6%, 95% CI: 0.008–0.026). The most prevalent types of FAs were found for milk and eggs (prevalence: 1.1%, 95% CI: 0.009–0.013 and 1.1%, 0.008–0.014, respectively). Breastfeeding (OR: 1.349, 95% CI: 1.011–1.799), male (OR: 1.289, 95% CI: 1.001–1.659), antibiotic exposure during pregnancy (OR: 1.221, 95% CI: 1.162–1.284), asthma (OR: 1.794, 95% CI: 1.443–2.230), eczema (OR: 5.121, 95% CI: 3.575–7.334), family history of atopic disease (OR: 1.893, 95% CI: 1.313–2.730), family history of atopic dermatitis (OR: 1.954, 95% CI: 1.645–2.322), family history of FAs (OR: 2.096, 95% CI: 1.686–2.594), family history of allergic rhinitis/conjunctivitis (OR: 1.287, 95% CI: 1.191–1.392, and family history of asthma (OR: 1.516, 95% CI: 1.370–1.678)) increased the risk of FA. The findings reveal that the prevalence of FA varies geographically, with Oceania having the highest frequency and Africa having the lowest prevalence. Exposure to antibiotics during pregnancy, being male, asthma, breastfeeding, eczema, FAs, family history of atopic disease, AD, allergic rhinitis/conjunctivitis, and asthma were all linked to an increased risk of FA [58]. The prevalence of FA varied by geographic location. To enhance the quality of life, FA-high-risk people should be better identified and cared for.

#### 3.4.6. Caesarean Section (CS)

Caesarean section (CS) has been shown to reduce the risk of maternal and perinatal mortality and morbidity [59]. However, the long-term risks and benefits of CS, especially in cases where there are no medical indications, are still not well understood. Research indicates that the composition of the microbiome during the fetal and neonatal stages plays a crucial role in immune system development. Babies born via CS have a distinct gut flora compared to those delivered vaginally, which may explain why CS is associated with an increased likelihood of allergic diseases later in life [60].

In line with our findings, a population-based birth cohort research involving 536 children looked at the link between CS and physician-diagnosed FA and found that children born through CS had a threefold increased risk of FA [34]. In another study, the experts found that children born by Caesarean section to moms who received intravenous antibiotics developed the same microbiota, but it was less plentiful [61] Based on 26 studies on delivery by C-section, the team found that vaginal delivery leads to the first colonization of the gut with maternal vaginal bacteria, whereas C-section babies are deprived of this natural exposure and have different gut flora, resulting in a 4% prevalence of moderately increased risk of FA/food atopy (OR 1.32, 95 percent CI 1.12–1.55) in 6 of their included studies [60,62]. In a meta-analysis that tried to quantify the difference in eczema prevalence between Caesarean-born and vaginal-born infants under 1 year of age, nine studies were included with 3758 participants who were born by Caesarean and 9631 individuals who were born vaginally. Compared to vaginal births (20.1%; 95% CI: 13.9–28.1), Caesarean births had a higher prevalence of eczema (27.8%; 95% CI: 17.7–39.2) with a pooled OR of 1.31 (95% CI: 1.04–1.65). In contrast to the 20% rate of occurrence reported in vaginally born infants, approximately 28% of infants born by Caesarean acquired eczema by the time they were 1 year old [63]. Another meta-analysis included 20,418 vaginal births and 9650 Caesarean births among children aged 0 to 3 years. There were 991 vaginally and 645 Caesarean-born children among them who had food allergies. The combined prevalence of food allergy was greater in infants born by Caesarean section (7.8%) than in those delivered vaginally (5.9%). An elevated risk of food allergy was shown to be linked to Caesarean sections [odds ratio (OR): 1.45; 95% confidence interval (CI): 1.03–2.05]. Additionally, children born via Caesarean delivery who had a parent with an allergy had a higher chance of developing a food allergy (OR: 2.60; 95% CI: 1.28–5.27) [64].

The transmission of the maternal microbiome is disrupted and the gut microbiome programming in the infant is compromised by Caesarean birth (C-section). Due to this, there is a higher chance that allergy disorders will emerge since it affects how the immune system develops [65]. The mother passes on her microbiome to the baby during vaginal birth, as the infant comes into contact with beneficial microbes present in the birth canal. However, this microbial transfer does not occur in babies delivered via C-section. Consequently, newborns delivered by C-section require additional time to establish a normal microbiota. Furthermore, due to the immaturity of their immune systems during this critical period, they are more susceptible to illnesses such as allergies [66]. The infant intestine’s colonization may take longer after a Caesarean birth. The rise in allergy illnesses has been attributed to a delayed or abnormal colonization process, which suggests that maternal microbes may be particularly significant due to several variables. First, compared to bacteria from other sources, it has been found that maternal microbes are more likely to successfully colonize the child’s bowel. Second, without the mother’s bacterial input, the newborn is more likely to become colonized by hospital germs, some of which are opportunistic and dangerous. Compared to children delivered vaginally, opportunistic microorganisms are more frequently found in children delivered after Caesarean section. These opportunistic microorganisms may harm the immune system in their own right. Third, studies have shown that Western babies have a lower bacterial turnover than babies from less developed nations [65].

#### 3.4.7. Antibiotics

The use of antibiotics in children has witnessed a notable surge in recent years that coincides with a substantial increase in the prevalence of allergic illnesses, especially food allergies. Extensive research has demonstrated a compelling association between the administration of antibiotics and the development of food allergies. For instance, a meta-analysis conducted on this subject matter discovered a statistically significant correlation between early-life antibiotic usage and an elevated risk of food allergies later in life. Specifically, it was found that children who were exposed to antibiotics during their first year faced a heightened likelihood of developing food allergies compared to those who did not receive antibiotics (OR: 1.42, 95% CI: 1.08–1.87; I2: 80.8%) [67].

Furthermore, the systematic review and meta-analysis included in this study revealed that four out of the six studies examined also demonstrated a relationship between early-life antibiotic exposure and childhood food allergies. Antibiotics and food allergies are likely linked through multiple mechanisms. One major pathway through which antibiotic exposure affects the likelihood of developing food allergies is the disruption of the microbiome. The microbiome plays a vital role in immune system development, making it a key factor in the development of allergies. Broad-spectrum antibiotics, in particular, have a greater impact on the microbiome compared to narrow-spectrum antibiotics, thus increasing the influence on immune system development and allergy susceptibility [68]. According to a meta-analysis, early exposure to antibiotics may raise the likelihood of developing hay fever, eczema, and food allergies in later life. The combined OR for the risk of hay fever (based on 22 trials) was 1.23, with a 95% confidence interval (CI) of 1.13–1.34 and an I2 of 77.0%. The summary odds ratio (OR) for the risk of eczema (22 studies) was 1.26, 95% confidence interval (CI): 1.15–1.37; I2: 74.2%, while the summary odds ratio (OR) for food allergy (3 studies) was 1.42, 95% CI: 1.08–1.87; I2: 80.8% [69]. Another meta-analysis includes a total of 26 studies. The results show that the pooled OR for food allergy indicated 1.36 (95% CI = 0.94–1.96), the overall OR for eczema/atopic dermatitis indicated 1.62 (95% CI = 1.16–2.27), and the summary OR for maternal antibiotic exposure during pregnancy was 1.29 (95% CI = 1.16–1.43). Antibiotic use by mothers during pregnancy may raise the likelihood that children will develop allergies [69].

Finally, this study explores a significant concept regarding the features of FA development. Building on prior research, it is concluded by the authors that adopting a varied diet along with a healthy lifestyle can effectively mitigate the occurrence of allergic reactions in the future [70]. Although limited empirical studies have been conducted on the multiple food strategy, there is already an emerging inclination towards this approach in the research field [71,72].

## 4. Limitations and Strengths

When extrapolating our findings to broader populations, readers need to consider the limitations of our study. Firstly, the studies and their outcomes were reliant on self-reported data. Secondly, a constraint of this study was the limited number of primary articles included, totaling only nine, and the exclusion of languages other than English, such as Spanish, Portuguese, Chinese, and Russian, which may have contained relevant articles. Consequently, the generalizability of our findings may be restricted due to insufficient supporting data in the field of FA. However, this study does possess certain strengths. It should be noted that underestimation cannot be entirely ruled out despite the possibility of overestimating the prevalence of self-reported food allergies. Nonetheless, this review presents a comprehensive overview of the published evidence on the impact of dietary and lifestyle changes on food allergies spanning from childhood to adulthood on a global scale. Our meticulous search strategy involved collaboration with experts in scoping review searches. This study is the first known effort to provide an overview the of existing research literature examining the effects of lifestyle and dietary habits on the prevalence of food allergies. While it is important to acknowledge that this review exclusively included studies written in English, potentially overlooking crucial FA research in other languages, our findings will contribute to a better understanding of the current state of FA prevalence and identify areas for further research.

## 5. Recommendation

After conducting this study, we recommend the following:

Health education: the general public must be informed about food allergens’ potential risk factors. More research has to be performed with bigger samples and more disparity.

## 6. Conclusions

Concerns have been raised regarding the frequency of food allergies (FAs) in the general population, particularly among patients and their families, healthcare providers, schools, food producers and merchants, and government agencies. While there are publications on the prevalence of FA, it remains uncertain whether reliable conclusions can be drawn about the consistency of these estimates and the quality of the data from these studies. This issue is urgent because, with few exceptions, there is a prevailing belief that the prevalence of FA is increasing, despite the lack of longitudinal data. Many papers estimate the incidence of FA solely based on reported food reactions, which may lead to an overestimation of the factors contributing to the increased prevalence of FA when compared to research utilizing objective diagnostic techniques. The impact of lifestyle and dietary choices on the development of allergies is a heavily debated topic. In our scoping review, we discovered that certain lifestyle factors (such as Caesarean section and antibiotic use) and dietary habits (including n-3 PUFA, fast food consumption, duration of dietary intervention, and vitamin D levels) are important factors in the development of food allergies. To gain a better understanding of the etiology of allergy disorders and potentially identify preventive interventions, it is crucial to conduct epidemiological investigations on well-defined research cohorts.

## Figures and Tables

**Table 1:** Useful components of the medical history [6].

Number	Question/Inquiry	Knowledge of Onset
1	Was one of the most common allergenic meals consumed two hours or less before the response started?	Most food allergies are caused by a small number of foods, including cow’s milk, eggs, peanuts, tree nuts, shellfish, finned fish, wheat, and soy.
2	Has there been prior consumption of the suspected food?	If you have previously tolerated a dish, it is less probable that this time will be different.
3	Has there been a previous response?	Reactions to the same food repeatedly indicate that it is more likely the cause.
4	What age did the food allergy start?	The most common causes of allergy onset in kids are allergies to milk, eggs, wheat, or peanuts, while the most common causes of allergy onset in adults are allergies to nuts, shellfish, or pollen–food allergy syndrome.
5	How was the food prepared?	Although reactions to small amounts of food can also happen, larger amounts of food are more likely to do so. For example, a person who is sensitive to whole cow’s milk or raw eggs may be able to handle smaller amounts of heated versions in baked products (such cookies or muffins).
6	Were there any enhancing influences?	Menstruation, exercise, infection, usage of pharmaceuticals (such as nonsteroidal anti-inflammatory drugs), and alcohol intake can increase responsiveness or make a reaction more severe.
7	What signs and symptoms did the patient experience?	IgE-mediated reactions are characterized by symptoms in the skin, lungs, stomach, and heart. An older person would feel “doom”, whereas a small child might weep, stop playing, or become listless.

**Table 2:** Keywords and subject headings used during the search.

Concept	Keyword	MeSH
**Concept 1**	Food hypersensitivity	“Food Hypersensitivity” [Mesh] OR “Food Hypersensitivity” OR “FA” OR Hypersensitivities OR “hypersensitivity” OR “allergic” OR “sensitivities” OR “Allergies” OR “Food Allergies” OR “food” OR “oral allergy syndrome”
**Concept 2**	Prevalence	“Prevalence” [Mesh] OR “Prevalence*” OR “epidemiology” OR “increase” OR “rise”
**Concept 3**	Lifestyle	“Life Style” [Mesh] “Lifestyle*” OR “behavior” OR “way of life” OR “condition” OR “situation”
**Concept 4**	Dietary habits	“Feeding Behavior” [Mesh] OR “Feeding Behavior” OR “diet” OR “feeding” OR “eating behavior” OR “consumption habit” OR “Feeding-Related Behaviors” OR “feeding pattern” OR “food habit”

**Table 3:** Inclusion and exclusion criteria for this review.

Factors	Inclusion Criteria	Exclusion Criteria
**Settings**	Any country; studies where specified data (see below) were collected with timeline between 2016 and 2021	Studies reporting on data collected before 2016
**Participants**	Any age	Participant groups selected based on a pre-existing health condition (including obesity, eating disorders, malnutrition)
**Outcomes**	A measure of diet and lifestyle (quantitative synthesis)	No link to the effect of lifestyle and diet on FAStudies reporting tracking of cigarette smokingStudies reporting solely alcohol intakeStudies reporting eating disorders or weight reduction behaviorsStudies reporting obesity
**Study Type**	Open access (cross-sectional studies, longitudinal prospective quantitative studies, with data reported including on specified outcome, human subjects)	Review papers, books, animal subjects, qualitative studies
**Publication type**	Journal article	Conference abstract, study protocol, report, thesis, dissertation, book, professional journal
**Publication Date**	2016–2020	Published date other than the selected date
**Language**	English	Languages other than English

**Table 4:** Characteristics and findings of included articles of included studies (*n* = 8).

Reference/Dates	Country	Sample Size and Age	Design of Publication	Study Limitations	Main Outcome
Papathoma et al., (2016) [34]	Greece	n = 459, 34 weeks	Prospective birth cohort study/Pediatric Allergy and Immunology	Data from a single center and a limited urban/suburban area. Small study sample. Lack of socioeconomical data. The proposed intermediate role of gut microbiota remains an assumption.	Delivery by CS predisposes the development of FAs but not atopic dermatitis in early childhood. Caesarean section delivery seems to upregulate the immune response to food allergens, especially in children with allergic predispositions.
Rosendahl et al., (2017) [35]	Finland	n = 171, 10 years	Cross-sectional study/Hormone Research in Paediatrics	This was a cross-sectional study with small N. Lacked information on consumed portion sizes and the use of vitamin-D-fortified fat spreads.	Fortification of food products with vitamin D have been successful in improving vitamin D status in children.
Van Zyl, (2018) [36]	South Africa	n = 250, women/mothers	Longitudinal observational research/Journal Article	The study’s limitations include a small sample size, the utilization of dietary intake data for zinc and vitamin E instead of assessing their actual status, and the inherent shortcomings associated with employing a Quantitative Food Frequency Questionnaire (QFFQ) to estimate accurate dietary intake.	A potential up-regulation of both fatty acid desaturase and elongase enzyme activity with a notable emphasis on elongase was observed. This finding predominantly highlights the involvement of the n-6 fatty acid pathway, suggesting a potential shift towards a more pro-inflammatory state.
Li et al., (2019) [37]	US	1,001294, 2–18 years	Cross-sectional study /Journal of Allergy and Clinical Immunology: In Practice	N/A	Recent research has established a robust association between the exposure to antibiotics and the development of food allergies.
Ribeiro et al., (2020) [10]	Australia	n = 5780, 5–12 years	Cross-sectional cohort study/Allergology International	Mothers reported the daily frequency of their children’s intake using a specialized tool known as the Children’s Dietary Questionnaire.	Asian children who were born in Australia exhibited a higher prevalence of nut allergy compared to Asian children who had immigrated to Australia.
Hicke-Robert et al., (2020) [38]	Sweden	n = 1838, 7–8 years	Cross-sectional studies/BMC Pediatrics	The response rate in this study was approximately 60%, which is a common level observed in con-temporary epidemiological studies.	Late introduction of solids into infants’ diets may increase the risk of food allergy development.
Cho et al., (2020) [39]	Korea	n = 53,373, 15.03 ± 1.75 years	Cross-sectional study/European Journal of Nutrition	The severity of food allergies (FAs) in individuals who had recently been diagnosed or previously diagnosed was assessed through a survey. However, the survey did not provide a means to evaluate the severity of FA. Additionally, evaluating long-term food intake proved challenging. Furthermore, it is important to consider that responses or expo-sures to food in different countries may vary due to racial or cultural differences.	Frequent intake of fast foods, energy drinks, and convenience food was related to recently diagnosed FA in adolescents.
Kayale et al., (2020) [40]	UK	n = 3300, 3–16 years	Cross-sectional study/PLoS ONE	The data collection for this study relied solely on a questionnaire, without any clinical data being collected. Consequently, the accuracy of the findings is dependent on the respondents’ ability to recall information accurately. It is well known that recall can be both biased and inaccurate, with a potential limitation of parents forgetting events from early childhood due to the wide age range of children (3–16 years) included in the study. Moreover, it is worth noting that the overall response rate was 34%.	Regular and early consumption of a moderate number of nuts during infancy, as well as maternal ingestion of nuts during pregnancy or while breastfeeding, is believed to support the development of tolerance.

**Table 5:** OR values of the included studies in the scoping review.

Factors	Author(s)	Publication Year	Factors	Country	Odd Ratio	Lower CI	Upper CI
Lifestyle	Papathoma et al. [34]	2016	C-section	Greece	3.15	1.14	8.7
	Rosendahl et al. [35]	2017	Vitamin D	Finland	0.14	0.039	0.241
	Hicker-Rober [38]	2020	Late introduction	Sweden	1.8	1.15	3.02
Dietary habits	Li et al. [37]	2019	Antibiotic	US	1.4	1.34	1.45
	Ribeiro et al. [10]	2018	Ultra-processed foods	Australia	1.28	1.08	1.51
	Van Zyl et al. [36]	2018	n-3 PUFA	South Africa	1.21	1.13	1.37
	Cho et al. [39]	2020	Ultra-processed foods	Korea	1.405	1.15	1.717
	Kayale et al. [40]	2020	Geographical Location	UK	1.0706	0.1108	10.3493

## Data Availability

The data presented in this study are available on request from the corresponding authors.

## References

[1] Kanchan K., Clay S., Irizar H., Bunyavanich S., Mathias R.A. (2021). Current insights into the genetics of food allergy. J. Allergy Clin. Immunol..

[2] Rona R.J., Keil T., Summers C., Gislason D., Zuidmeer L., Sodergren E., Sigurdardottir S.T. (2006). The prevalence of food allergy: A meta-analysis General approach. 2006, 120, 638–646. J. Allergy Clin. Immunol..

[3] Jackson C.M., Mahmood M.M., Järvinen K.M. (2021). Farming lifestyle and human milk: Modulation of the infant microbiome and protection against allergy. Acta Paediatr..

[4] Renz H., Allen K.J., Sicherer S.H., Sampson H.A., Lack G., Beyer K., Oettgen H.C. (2018). Food allergy. Nat. Rev. Dis. Prim..

[5] Mendes C., Costa J., Vicente A.A., Oliveira M.B.P.P., Mafra I. (2019). Cashew Nut Allergy: Clinical Relevance and Allergen Characterisation. Clin. Rev. Allergy Immunol..

[6] Oriel R.C., Wang J. (2019). Diagnosis and Management of Food Allergy. Pediatr. Clin. N. Am..

[7] Laia-Dias I., Lozoya-Ibáñez C., Skypala I., Gama J.M.R., Nurmatov U., Lourenço O., Taborda-Barata L. (2019). Prevalence and risk factors for food allergy in older people: Protocol for a systematic review. BMJ Open.

[8] Hoi A.Y., Ross L., Day J., Buchanan R.R.C. (2017). Immunotherapeutic strategies in antiphospholipid syndrome. Intern. Med. J..

[9] Cosme-Blanco W., Arroyo-Flores E., Ale H. (2020). Food allergies. Pediatr. Rev..

[10] Ribeiro L.W., Moss K.M., Mishra G.D. (2020). Dietary patterns and allergy in children aged 5–12 years in Australia: Findings from the Mothers and Their Children’s Health study. Allergol. Int..

[11] Krifa I., Hallez Q., van Zyl L.E., Braham A., Sahli J., Ben Nasr S., Shankland R. (2021). Effectiveness of an online positive psychology intervention among Tunisian healthcare students on mental health and study engagement during the COVID-19 pandemic. Appl. Psychol. Health Well-Being.

[12] Benedé S., Blázquez A.B., Chiang D., Tordesillas L., Berin M.C. (2016). The rise of food allergy: Environmental factors and emerging treatments. EBioMedicine.

[13] Lopes J.P., Sicherer S. (2020). Food allergy: Epidemiology, pathogenesis, diagnosis, prevention, and treatment. Curr. Opin. Immunol..

[14] Savage J., Johns C.B. (2015). Food Allergy Epidemiology and Natural History Food allergy Epidemiology Natural history Peanut Milk Egg. Immunol. Allergy Clin..

[15] Stemeseder T., Klinglmayr E., Moser S., Lang R., Himly M., Oostingh G.J., Zumbach J., Bathke A.C., Hawranek T., Gadermaier G. (2017). Influence of Intrinsic and Lifestyle Factors on the Development of IgE Sensitization. Int. Arch. Allergy Immunol..

[16] Wang J., Vanga S.K., Raghavan V. (2019). Effect of pre-harvest and post-harvest conditions on the fruit allergenicity: A review. Crit. Rev. Food Sci. Nutr..

[17] Anvari S., Miller J., Yeh C., Davis C.M. (2019). IgE-Mediated Food Allergy. Clin. Rev. Allergy Immunol..

[18] Sathe S.K., Liu C., Zaffran V.D. (2016). Food Allergy. Annu. Rev. Food Sci. Technol..

[19] Satitsuksanoa P., Jansen K., Głobińska A., van de Veen W., Akdis M. (2018). Regulatory immune mechanisms in tolerance to food allergy. Front. Immunol..

[20] Reynolds L.A., Finlay B.B. (2017). Early life factors that affect allergy development. Nat. Rev. Immunol..

[21] Sicherer S.H., Sampson H.A. (2010). Food allergy. J. Allergy Clin. Immunol..

[22] Sicherer S.H., Sampson H.A. (2018). Food allergy: A review and update on epidemiology, pathogenesis, diagnosis, prevention, and management. J. Allergy Clin. Immunol..

[23] Yu W., Freeland D., Nadeau K. (2016). Food allergy: Immune mechanisms, diagnosis and immunotherapy. Nat. Rev. Immunol..

[24] Boyce J.A. (2010). Guidelines for the diagnosis and management of food allergy in the United States: Report of the NIAID-sponsored expert panel. J. Allergy Clin. Immunol..

[25] Gupta R.S., Warren C.M., Smith B.M., Jiang J., Blumenstock J.A., Davis M.M., Schleimer R.P., Nadeau K.C. (2019). Prevalence and Severity of Food Allergies Among US Adults. JAMA Netw. Open.

[26] Wang J., Vanga S.K., McCusker C., Raghavan V. (2019). A Comprehensive Review on Kiwifruit Allergy: Pathogenesis, Diagnosis, Management, and Potential Modification of Allergens Through Processing. Compr. Rev. Food Sci. Food Saf..

[27] Messina M., Venter C. (2020). Recent Surveys on Food Allergy Prevalence. Nutr. Today.

[28] Pacific A. (2012). Asia Pacific allergy. Asia Pac. Allergy.

[29] Tricco A.C., Lillie E., Zarin W., O’Brien K.K., Colquhoun H., Levac D., Moher D., Peters M.D., Horsley T., Weeks L. (2018). PRISMA extension for scoping reviews (PRISMA-ScR): Checklist and explanation. Ann. Intern. Med..

[30] Peters M.D., Godfrey C., McInerney P., Munn Z., Tricco A.C. (2020). Chapter 11: Scoping reviews. JBI Manual for Evidence Synthesis.

[31] Malley O. (2005). Scoping studies: Towards a methodological framework. Int. J. Soc. Res. Methodol..

[32] Levac D., Colquhoun H., O’Brien K.K. (2010). Scoping studies: Advancing the methodology. Implement. Sci..

[33] McGowan J., Straus S., Moher D., Langlois E.V., O’Brien K.K., Horsley T., Aldcroft A., Zarin W., Garitty C.M., Hempel S. (2020). Reporting scoping reviews—PRISMA ScR extension. J. Clin. Epidemiol..

[34] Papathoma E., Triga M., Fouzas S. (2016). Cesarean section delivery and development of food allergy and atopic dermatitis in early childhood. Pediatr. Allergy Immunol..

[35] Rosendahl J., Fogelholm M., Pelkonen A. (2017). A History of Cow’s Milk Allergy Is Associated with Lower Vitamin D Status in Schoolchildren. Horm. Res. Paediatr..

[36] Van Zyl I. (2018). Fatty Acid and Micronutrient Intake and Status in Association with Allergy among Pregnant Urban Women in South Africa. Ph.D. Thesis.

[37] Li M., Lu Z.K., Amrol D.J., Mann J.R., Hardin J.W., Yuan J., Cox C.L., Love B.L. (2019). Antibiotic Exposure and the Risk of Food Allergy: Evidence in the US Medicaid Pediatric Population. J. Allergy Clin. Immunol. Pract..

[38] Hicke-Roberts A., Wennergren G., Hesselmar B. (2020). Late introduction of solids into infants’ diets may increase the risk of food allergy development. BMC Pediatr..

[39] Cho S.I., Lee H., Lee D.H., Kim K.H. (2020). Association of frequent intake of fast foods, energy drinks, or convenience food with atopic dermatitis in adolescents. Eur. J. Nutr..

[40] Kayale L.B., Ling J., Henderson E., Carter N. (2020). The influence of cultural attitudes to nut exposure on reported nut allergy: A pilot cross sectional study. PLoS ONE.

[41] Annesi-Maesano I., Fleddermann M., Hornef M., von Mutius E., Pabst O., Schaubeck M., Fiocchi A. (2021). Allergic diseases in infancy: I—Epidemiology and current interpretation. World Allergy Organ. J..

[42] Best K.P., Gold M., Kennedy D., Martin J., Makrides M. (2016). Omega-3 long-chain PUFA intake during pregnancy and allergic disease outcomes in the offspring: A systematic review and meta-analysis of observational studies and randomized controlled trials. Am. J. Clin. Nutr..

[43] Bärebring L., Nwaru B.I., Lamberg-Allardt C., Thorisdottir B., Ramel A., Söderlund F., Arnesen E.K., Dierkes J., Åkesson A. (2022). Supplementation with long chain n-3 fatty acids during pregnancy, lactation, or infancy in relation to risk of asthma and atopic disease during childhood: A systematic review and meta-analysis of randomized controlled clinical trials. Food Nutr. Res..

[44] Wang C.S., Wang J., Zhang X., Zhang L., Zhang H.P., Wang L., Wood L.G., Wang G. (2018). Is the consumption of fast foods associated with asthma or other allergic diseases?. Respirology.

[45] Melo B., Rezende L., Machado P., Gouveia N., Levy R. (2018). Associations of ultra-processed food and drink products with asthma and wheezing among Brazilian adolescents. Pediatr. Allergy Immunol..

[46] Hoffam R. (2022). Ultra-Processed Foods Are Harmful for Our Health—Here’s Why. The Conversation. https://interestingengineering.com/health/ultra-processed-food-harmful.

[47] Kakieu Djossi S., Khedr A., Neupane B., Proskuriakova E., Jada K., Mostafa J.A. (2022). Food Allergy Prevention: Early Versus Late Introduction of Food Allergens in Children. Cureus.

[48] Olenec J., Gern J.E. (2009). Age at first introduction of cow milk products and other food products in relation to infant atopic manifestations in the first 2 years of life: The KOALA birth cohort study. Pediatrics.

[49] Nwaru B.I., Erkkola M., Ahonen S., Kaila M., Haapala A.M., Kronberg-Kippilä C., Salmelin R., Veijola R., Ilonen J., Simell O. (2010). Age at the introduction of solid foods during the first year and allergic sensitization at age 5 years. Pediatrics.

[50] Dai N., Li X., Wang S., Wang J., Gao Y., Li Z. (2021). Timing of food introduction to the infant diet and risk of food allergy: A systematic review and Meta-analysis. J. Chin. Med. Assoc..

[51] Smith P.K., Masilamani M., Li X.-M., Sampson H.A. (2017). The false alarm hypothesis: Food allergy is associated with high dietary advanced glycation end-products and proglycating dietary sugars that mimic alarmins. J. Allergy Clin. Immunol..

[52] Vassallo M.F., Camargo C.A. (2010). Potential mechanisms for the hypothesized link between sunshine, vitamin D, and food allergy in children. J. Allergy Clin. Immunol..

[53] Lieberman J.A., Greenhawt M., Nowak-Wegrzyn A. (2018). The environment and food allergy. Ann. Allergy Asthma Immunol..

[54] Allen K.J., Koplin J.J., Ponsonby A.L., Gurrin L.C., Wake M., Vuillermin P., Martin P., Matheson M., Lowe A., Robinson M. (2013). Vitamin D insufficiency is associated with challenge-proven food allergy in infants. J. Allergy Clin. Immunol..

[55] Willits E.K., Wang Z., Jin J., Patel B., Motosue M., Bhagia A., Almasri J., Erwin P.J., Kumar S., Joshi A.Y. (2017). Vitamin D and food allergies in children: A systematic review and meta-analysis. Allergy Asthma Proc..

[56] Psaroulaki E., Katsaras G.N., Samartzi P., Chatziravdeli V., Psaroulaki D., Oikonomou E., Tsitsani P. (2023). Association of food allergy in children with vitamin D insufficiency: A systematic review and meta-analysis. Eur. J. Pediatr..

[57] Panjari M., Koplin J.J., Dharmage S.C., Peters R.L., Gurrin L.C., Sawyer S.M., Mcwilliam V., Eckert J.K., Vicendese D., Erbas B. (2016). Nut allergy prevalence and differences between Asian-born children and Australian-born children of Asian descent: A state-wide survey of children at primary school entry in Victoria, Australia. Clin. Exp. Allergy.

[58] Feng H., Xiong X., Chen Z., Xu Q., Zhang Z., Luo N., Wu Y. (2023). Prevalence and Influencing Factors of Food Allergy in Global Context: A Meta-Analysis. Int. Arch. Allergy Immunol..

[59] Thomas S., Meadows J., McQueen K.A.K. (2016). Access to Cesarean Section Will Reduce Maternal Mortality in Low-Income Countries: A Mathematical Model. World J. Surg..

[60] Gu L., Zhang W., Yang W., Liu H. (2019). Systematic review and meta-analysis of whether cesarean section contributes to the incidence of allergic diseases in children: A protocol for systematic review and meta-analysis. Medicine.

[61] Sbihi H., Boutin R.C.T., Cutler C., Suen M., Finlay B.B., Turvey S.E. (2019). Thinking bigger: How early-life environmental exposures shape the gut microbiome and influence the development of asthma and allergic disease. Allergy Eur. J. Allergy Clin. Immunol..

[62] Bager P., Wohlfahrt J., Westergaard T. (2008). Caesarean delivery and risk of atopy and allergic disesase: Meta-analyses. Clin. Exp. Allergy.

[63] Xiong Z., Zhou L., Chen Y., Wang J., Zhao L., Li M., Chen I., Krewski D., Wen S.W., Xie R. (2022). Prevalence of eczema between cesarean-born and vaginal-born infants within 1 year of age: A systematic review and meta-analysis. Eur. J. Pediatr..

[64] Yang X., Zhou C., Guo C., Wang J., Chen I., Wen S.W., Krewski D., Yue L., Xie R.H. (2023). The prevalence of food allergy in cesarean-born children aged 0–3 years: A systematic review and meta-analysis of cohort studies. Front. Pediatr..

[65] Loo E.X.L., Sim J.Z.T., Loy S.L., Goh A., Chan Y.H., Tan K.H., Yap F., Gluckman P.D., Godfrey K.M., Van Bever H. (2017). Europe PMC Funders Group Associations between caesarean delivery and allergic outcomes: Results from the GUSTO study. Ann. Allergy Asthma Immunol..

[66] Verbanas P. C-Section Delivery Prevents Babies from Receiving Beneficial Germs in Their Mother’s Microbiome, Which, in Turn, Affects Immune System Development, Says Rutgers Researcher. Rutgers University. https://www.rutgers.edu/news/hidden-reason-children-born-c-section-are-more-likely-develop-asthma.

[67] Ahmadizar F., Vijverberg S.J.H., Arets H.G.M., de Boer A., Lang J.E., Garssen J., Kraneveld A., Maitland-van der Zee A.H. (2018). Early-life antibiotic exposure increases the risk of developing allergic symptoms later in life: A meta-analysis. Allergy Eur. J. Allergy Clin. Immunol..

[68] Netea S.A., Messina N.L., Curtis N. (2019). Early-life antibiotic exposure and childhood food allergy: A systematic review. J. Allergy Clin. Immunol..

[69] Zhong Y., Zhang Y., Wang Y., Huang R. (2021). Maternal antibiotic exposure during pregnancy and the risk of allergic diseases in childhood: A meta-analysis. Pediatr. Allergy Immunol..

[70] Perkin M.R., Logan K., Tseng A., Raji B., Ayis S., Peacock J., Brough H., Marrs T., Radulovic S., Craven J. (2016). Randomized Trial of Introduction of Allergenic Foods in Breast-Fed Infants. N. Engl. J. Med..

[71] Cukrowska B. (2018). Microbial and nutritional programming—The importance of the microbiome and early exposure to potential food allergens in the development of allergies. Nutrients.

[72] D’Auria E., Peroni D.G., Sartorio M.U.A., Verduci E., Zuccotti G.V., Venter C. (2020). The Role of Diet Diversity and Diet Indices on Allergy Outcomes. Front. Pediatr..

